# Foot insensitivity is associated with renal function decline in patients with type 2 diabetes: a cohort study

**DOI:** 10.1186/s12902-016-0147-1

**Published:** 2016-11-22

**Authors:** Quratul A. Altaf, Hamed Sadiqi, Milan K. Piya, Abd A. Tahrani

**Affiliations:** 1 Department of Diabetes and Endocrinology, Heart of England NHS Foundation Trust, Birmingham, UK; 2 Derby Teaching Hospitals NHS Foundation Trust, Derby, UK; 3 Warwick Medical School, University of Warwick, Coventry, UK; 4 Institute of Metabolism and Systems, School of Clinical and Experimental Medicine, University of Birmingham, Birmingham, UK; 5 Centre of Endocrinology, Diabetes and Metabolism, Birmingham Health Partners, Birmingham, B15 2TT UK

**Keywords:** Diabetic neuropathy, Chronic kidney disease, Diabetic nephropathy, Albuminuria, Estimated glomerular filtration rate, Foot insensitivity, 10 g monofilament

## Abstract

**Background:**

Identifying patients with diabetes at increased risk of chronic kidney disease (CKD) is essential to prevent/slow the progression to end-stage renal disease (ESRD). CKD and diabetic peripheral neuropathy (DPN) share common mechanisms. Hence, we aimed to examine the relationship between foot insensitivity and CKD in patients with Type 2 diabetes.

**Methods:**

A prospective observational cohort study in adults with Type 2 diabetes. Patients with ESRD were excluded. Foot insensitivity was assessed using the 10-g monofilament test. Renal function was assessed using estimated glomerular filtration rate (eGFR) based on the MDRD equation. Albuminuria was defined as the presence of urinary albumin/creatinine ratio (ACR) >3.4 mg/mmol.

**Results:**

Two hundred and twenty eight patients were recruited and followed-up for 2.5 years. One hundred and ninety patients (83.4%) had eGFR ≥ 60 ml/min/1.73 m^2^. Seventy six (33.3%) patients had foot insensitivity (i.e. abnormal monofilament test). Patients with foot insensitivity had lower eGFR and higher prevalence of albuminuria compared to patients with normal monofilament test. After adjustment for age, gender, ethnicity, diabetes duration, HbA1c, body mass index, insulin treatment, number of anti-hypertensives, history of peripheral vascular disease, and baseline eGFR (R^2^ 0.87), baseline foot insensitivity was associated with study-end eGFR (B = −3.551, *p* = 0.036).

**Conclusions:**

Patients with Type 2 diabetes and foot insensitivity are at increased risk of eGFR decline. Identifying these patients offers an opportunity to intensify metabolic and blood pressure control to prevent/retard the development of CKD. Future studies of larger sample size and longer follow up from multiple centres are needed to assess the diagnostic performance of our findings in predicting CKD development, and to compare the performance of the monofilament test with albuminuria.

## Background

Chronic kidney disease (CKD) in diabetes is the most common cause of end-stage renal disease (ESRD) and is associated with high morbidity and mortality [1, 2]. CKD in patients with diabetes progresses slowly, often starting with microalbuminuria, which progresses to overt proteinuria in 20–40% of patients and overall 20% of patients will have progressed to ESRD within 20 years after onset of overt proteinuria [1]. The speed of CKD progression is variable and largely dependent on blood pressure (BP), obesity, metabolic control and other factors such age, male sex and ethnicity [3, 4].

The pathogenesis of CKD is thought to be similar to other microvascular complications and includes major roles for hyperglycemia and hypertension promoting increased oxidative and nitrosative stress and the activation of multiple pathways that lead to increased inflammation and endothelial dysfunction [3]. In addition, hemodynamic changes occur as a result of the activation of the renin–angiotensin–aldosterone (RAAS) and endothelin systems, resulting in increased systemic and intra-glomerular pressure, causing hyperfiltration and albuminuria [3]. Despite attempts to improve metabolic control and RAAS inhibition, CKD remains very common and many patients develop ESRD requiring renal replacement therapy (RRT). Currently, albuminuria is widely used to predict progression to ESRD but eGFR decline may be the only manifestation of CKD progression in patients with diabetes without any evidence of albuminuria in up to 30% of cases [5, 6].

In longitudinal studies of patients with type 2 diabetes, we have previously identified obstructive sleep apnoea (OSA) and cardiac autonomic neuropathy (CAN) as novel predictors of eGFR decline [7, 8]. Clinically evident DPN is common in patients with CAN and in patients with OSA [9, 10] and as diabetic peripheral neuropathy (DPN) shares many aspects of its pathogenesis with CKD, we hypothesised that foot insensitivity may predict changes in renal function in patients with Type 2 diabetes.

Hence, we have conducted a study to assess the impact of foot insensitivity on eGFR in patients with Type 2 diabetes. A secondary outcome was to assess the impact of foot insensitivity on the development of albuminuria in patients with Type 2 diabetes.

## Methods

We conducted a prospective observational cohort study in White European and South Asian adults with type 2 diabetes. Patients were recruited between 2009 and 2010 and were followed until the end of 2012. Patients with ESRD or significant chronic respiratory disorder were excluded. Patients were recruited consecutively from the diabetes clinics of a UK-based hospital (Birmingham Heartlands Hospital). Patients were approached consecutively in the waiting area by the investigator or a research nurse without any prior knowledge of their medical condition. Consent was obtained and ethnicity determined in accordance with the UK decennial census by the study participants. The study was approved by the Warwickshire Research Ethics Committee (REC number 08/H1211/145) and funded by the National Institute for Health Research (NIHR) in the UK. Previous publications related to this study population can be found in [7, 8, 10, 11].

Foot insensitivity was defined as < 8 correct responses to a 10-g monofilament applied to 10 foot locations [12]. The sensitivity and specificity of the monofilament test to predict amputations or foot ulceration were 62 and 92% respectively [12]. Monofilament insensitivity was found to be an independent predictor of foot ulceration and amputations [13–15].

eGFR was calculated using the 4-variable MDRD equation [16]. The urinary albumin creatinine ratio (ACR) of a single early morning urine measurement was used to assess albuminuria as we have previously described [8]. Microalbuminuria was defined as ACR > 3.4 mg/mmol and macroalbuminuria was defined as ACR ≥ 30 mg/mmol [17–19]. Urine samples with evidence of urinary tract infection were repeated when free from infection. ACR and eGFR were measured at baseline and study-end. Study-end measurements were taken during patient visits for follow-up appointments at the diabetes clinic. eGFR and ACR measurements during acute illness or following imaging using contrast or from patients with significant haematuria, or proteinuria without albuminuria were excluded.

All assessments in the study (renal function and biochemical profiles) were conducted blinded to foot insensitivity status.

### Outcome measures and analyses

Data analysis was performed using SPSS 21.0 software (SPSS Inc, Chicago, USA). Data distribution was examined using histograms and the Shapiro-Wilk test. Data are presented as mean (SD) or median (IQR). Independent continuous variables were compared using the Student’s *t*-test or the Mann-Whitney test. Categorical variables were compared using the Chi-squared test.

To assess the impact of foot insensitivity on eGFR decline and albuminuria progression, only patients with baseline and study-end measurements were used. For the albuminuria progression analysis only patients free of albuminuria at baseline were included. To account for baseline differences, linear (for continuous outcomes) and logistic (for dichotomous outcomes) regression were used. Variables included in both the logistic and linear regression models were based on those that differed between patients with and without foot insensitivity and the baseline value for outcome measure. The outcome measures in the longitudinal analysis were the study-end eGFR and progression to albuminuria. The “enter” method was used in the regression analysis.

Residuals and collinearity were considered in assessing fit of models to data. Sequentially removing variables involved in multicollinearity had limited impact on model estimates for the main exposure. Hence, final models presented thus include variables based on those that differed between patients with and without foot insensitivity, regardless of the presence of collinearity.

A *p*-value < 0.05 was considered significant in all statistical testing.

## Results

Two hundred and twenty eight (228) patients were recruited. Data regarding eGFR and the monofilament status were available in all patients at baseline. Data regarding albuminuria were available in 215 (out of 228, 94.3%) patients at baseline. Follow up eGFR and albuminuria data were available in 225 (out of 228, 98.7%), and 184 (out of 228, 80.1%) patients respectively. Missing data during the follow up were not available on the patients electronic records. The follow-up duration was similar in patients with and without foot insensitivity (2.5 ± 0.6 vs. 2.5 ± 0.7 years, *p* = 0.4). For eGFR, 47.4% (*n* = 108), 36.0% (*n* = 82), 15.4% (*n* = 35) and 1.3% (*n* = 3) had values ≥ 90, 60–89, 30–59 and 15–29 ml/min/1.73 m^2^ respectively.

### Baseline analysis

Baseline demographics in relation to monofilament status are summarised in Table 1.

**Table 1:** Baseline demographics in relation to foot insensitivity status

	Monofilament¯	Monofilament^+^	*P*
n	152	76	
Age (years)	54.9 ± 11.9	61.7 ± 11.2	<0.001
White Europeans	56 (36.8%)	46 (60.5%)	0.001
Male	80 (52.6%)	52 (68.4%)	0.023
Alcohol (drinks alcohol)	28 (18.4%)	17 (22.4%)	0.480
Smoking (never smoked)	98 (64.5%)	42 (55.3%)	0.178
Diabetes Duration (years)	10.9 ± 6.6	15.9 ± 8.5	<0.001
HBA1c (%)	8.1 ± 1.4	8.5 ± 1.6	0.045
Total cholesterol (mmol/l)	3.9 ± 1.0	3.9 ± 1.0	0.824
Triglycerides	2.0 ± 1.2	2.1 ± 1.3	0.605
Systolic BP (mmHg)	129.3 ± 15.4	131.7 ± 20.3	0.313
Diastolic BP (mmHg)	79.1 ± 9.7	76.3 ± 11.5	0.051
Mean arterial pressure	95.8 ± 10.2	94.8 ± 12.9	0.490
BMI (kg/m2)	32.7 ± 6.8	35.4 ± 10.4	0.045
Diabetic retinopathy (%)^a^	88 (58.7%)	59 (80.8%)	0.001
Foot ulceration (%)	1 (0.7%)	7 (9.2%)	0.002
IHD (%)	26 (17.1%)	20 (26.3%)	0.102
PVD (%)	4 (2.6%)	10 (13.2%)	0.002
Diabetic retinopathy (%)	88 (58.7%)	59 (80.8%)	0.001
Medication			
Calcium Antagonist	36 (23.7%)	30 (39.5%)	0.013
Beta Blocker	30 (19.7%)	21 (27.6%)	0.177
Alpha Blocker	6 (3.9%)	13 (17.1%)	0.001
Diuretic	38 (25.0%)	36 (47.4%)	0.001
RAAS inhibitors	105 (69.1%)	53 (69.7%)	0.919
Anti hypertensives	116 (76.3%)	66 (86.8%)	0.062
No. of antihypertensives	1.0 (1.0–2.0)	2.0 (1.0–3.0)	0.001^a^
Lipid lowering treatment	129 (84.9%)	64 (84.2%)	0.897
Incretin-based treatment	28 (18.4%)	9 (11.8%)	0.204
OAD	142 (93.4%)	69 (90.8%)	0.476
Insulin	70 (46.1%)	50 (65.8%)	0.005

*BMI* body mass index, *RAAS* renin–angiotensin–aldosterone system, *OAD* oral anti diabetes agents, *IHD* ischaemic heart disease, *PVD* peripheral vascular disease. Monofilament¯: normal 10 g monofilament test; Monofilament^+^: abnormal 10 g monofilament test i.e. foot insensitivity present

^a^Diabetic retinopathy data were available in 223 patients (150 with normal and 73 abnormal monofilament test). PVD, IHD and retinopathy data were based on patients electronic records

Patients with foot insensitivity (i.e. abnormal monofilament test) were older, had longer diabetes duration, were more obese, had higher HbA1c and had more anti-hypertensives compared to patients with normal monofilament test. Foot insensitivity was more common in White Europeans than South Asians and more patients with foot insensitivity had history of peripheral vascular disease (PVD) and were prescribed insulin compared to those with normal monofilament test.

The relationship between foot insensitivity and eGFR and albuminuria is summarised in Table 2. Patients with foot insensitivity had lower eGFR values and higher prevalence of albuminuria. There was no significant association between foot insensitivity and macroalbuminuria.

**Table 2:** Foot insensitivity and eGFR and albuminuria: Baseline analysis

Variable	Monofilament¯ *N* = 152^a^	Monofilament^+^ *N* = 76^a^	*p* value
eGFR (ml min^−1^ 1.73 m^−2^)	89.5 ± 26.3	79.9 ± 26.0	0.010
Albuminuria	42 (29.2%)	34 (47.9%)	0.007
Macroalbuminuria	13 (9.0%)	11 (15.5%)	0.157
eGFR (ml min^−1^ 1.73 m^−2^)			
≥ 90	78 (51.3%)	30 (39.5%)	0.159
60–89	54 (35.5%)	28 (36.8%)	
30–59	18 (11.8%)	17 (22.4%)	
15–29	2 (1.3%)	1 (1.3%)	

Monofilament¯: normal 10 g monofilament test

Monofilament^+^: abnormal 10 g monofilament test i.e. foot insensitivity present

^a^ *Albuminuria data was available in 144 patients without and 71 with foot insensitivity*

### Foot insensitivity and eGFR and albuminuria: follow-up analysis

Data regarding the progression of eGFR, albuminuria, and macroalbuminuria were available in 150 vs. 75, 83 vs. 29, and 112 vs. 50 patients without vs. with foot insensitivity respectively.

The impact of foot insensitivity on albuminuria and eGFR longitudinally is summarised in Tables 3 and 4. A higher proportion of patients with foot insensitivity at baseline progressed to develop albuminuria and macroalbuminuria over the follow-up period compared to patients with normal monofilament test. The eGFR decline was also greater in patients with abnormal vs. normal monofilament test. The impact of foot insensitivity on eGFR and the progression of albuminuria and macroalbuminuria were more pronounced in South Asians than White Europeans (Table 3).

**Table 3:** The impact of foot insensitivity on eGFR and progression to albuminuria longitudinally

Variable	Monofilament¯	Monofilament^+^	*p* value
Progression to albuminuria (N^a^ = 83 vs. 29)	11 (13.3%)	10 (34.5%)	0.012
Progression to macroalbuminuria (N^a^ = 112 vs. 50)	2 (1.8%)	7 (14.0%)	0.002
eGFR change (ml min^−1^ 1.73 m^−2^) (N^a^ = 150 vs. 75)	−2.0 (−9.0 to 3.0)	−7.0 (−17.0 to −2.0)	0.001
eGFR change as % of baseline eGFR (%) (N^a^ = 150 vs. 75)	−3.0 (−10.0 to 3.6)%	−9.5 (−18.5 to −2.5)%	<0.001
South Asians			
Progression to albuminuria (N^a^ = 50 vs. 8)	5 (10.0%)	3 (37.5%)	0.071
Progression to macroalbuminuria (N^a^ = 67 vs. 20)	0 (0.0%)	4 (20.0%)	0.002
eGFR change (ml min^−1^ 1.73 m^−2^) (N^a^ = 94 vs. 29)	−3.0 (−9.0 to 3.3)	−10.0 (−17 to −3.0)	0.001
eGFR change as % of baseline eGFR (%) (N^a^ = 94 vs. 29)	−2.9 (−10.7 to 3.6)%	−12.2 (−19.0 to −4.4)	<0.001
White Europeans			
Progression to albuminuria (N^a^ = 33 vs. 21)	6 (18.2%)	7 (33.3%)	0.204
Progression to macroalbuminuria (N^a^ = 45 vs. 30)	2 (4.4%)	3 (10.0%)	0.383
eGFR change (ml min^−1^ 1.73 m^−2^) (N^a^ = 56 vs. 46)	−2.0 (−8.0 to 2.0)	−5.5.0 (−15.0 to 1.3)	0.125
eGFR change as % of baseline eGFR (%) (N^a^ = 56 vs. 46)	−3.0 (−9.7 to 4.2)%	−7.7 (−18.6 to −2.5)%	0.091

Monofilament¯: normal 10 g monofilament test; Monofilament^+^: abnormal 10 g monofilament test i.e. foot insensitivity present

^a^For Monofilament¯ vs. Monofilament^+^ respectively

**Table 4:** The linear regression model for the association between foot insensitivity and study-end eGFR

Variable	Unstandardized Regression coefficients	*P* value
Baseline eGFR (ml min^−1^ 1.73 m^−2^)	0.893	<0.001
Age (years)	−0.078	0.327
Diabetes duration (years)	−0.106	0.339
Gender (men vs. women)	2.075	0.180
HBA1c (%)	−0.516	0.328
BMI (Kg/m^2^)	−0.126	0.233
Insulin treatment (yes vs. no)	−1.472	0.354
PVD (yes vs. no)	−10.044	0.001
Number of anti hypertensives	−0.528	0.399
Ethnicity (South Asians vs. White Europeans)	1.433	0.389
Monofilament test (abnormal vs. normal)	−3.551	0.036

R for the model = 0.931, R^2^ for the model = 0.866. The regression was performed using the “Enter” method. *eGFR* estimated glomerular filtration rate, *BMI* Body Mass Index, *PVD* Peripheral vascular disease; abnormal monofilament test = foot insensitivity present

After adjusting for age, gender, ethnicity, diabetes duration, HbA1c, BMI, insulin treatment, number of anti-hypertensives, history of PVD, and baseline eGFR (R^2^ 0.87), baseline foot insensitivity was associated with study-end eGFR (B = −3.551, *p* = 0.036) (Table 4). Other independent predictors of study-end eGFR were baseline eGFR (B = 0.893, *p* < 0.001) and history of PVD (B = −10.044, *p* = 0.001) (Table 4). Using change in eGFR between baseline and study-end as the outcome measure instead of study-end eGFR showed similar results (R^2^ 0.161).

After adjustment for age, gender, ethnicity, diabetes duration, HbA1c, BMI, insulin treatment, number of anti-hypertensives, and history of PVD (Nagelkerke R^2^ 0.328), baseline foot insensitivity was not significantly associated with progression to albuminuria (OR 2.998, 95% CI 0.79–11.38, *p* = 0.107).

Using the last mentioned model but using progression to macroalbuminuria as the outcome measure (Nagelkerke R^2^ 0.499), showed that foot insensitivity was not associated with the outcome (OR 9.37, 95%CI 0.93–94.61, *p* = 0.058). HbA1c and PVD were significant predictors of the progression to macroalbuminuria.

## Discussion

To our knowledge, this is the first study to examine the relationship between foot insensitivity and the progression of eGFR and albuminuria in patients with Type 2 diabetes. Our results show that foot insensitivity was associated with the decline in eGFR in patients with Type 2 diabetes.

Whilst there appear to be no plausible mechanisms by which foot insensitivity could have a direct/causal impact on CKD in patients with diabetes, the two complications share common pathophysiological mechanisms. These include increased oxidative stress, inflammation and endothelial dysfunction. They also, share many risk factors such as obesity, diabetes duration, older age, and hypertension [3, 20, 21]. Hence, it is not surprising that these complications commonly co-exist, and this is supported by the findings of our cross-sectional analysis. In addition, DPN and CAN commonly co-exist and we have shown previously that CAN predicts the eGFR decline in patients with Type 2 Diabetes [7, 9]. We have also previously shown an association between OSA and foot insensitivity, and that OSA predicts eGFR decline in patients with Type 2 diabetes [8, 10]. Hence, the observed relationships between DPN and eGFR in our study could be mediated, at least in part, by the relationships between DPN and OSA and CAN.

In this study, we found that while foot insensitivity was associated with changes in eGFR, it did not predict the development of albuminuria. This could be related to sample size as suggested by the odds ratios, but it could also be that foot insensitivity identifies patients with more advanced disease as the 10 g monofilament is a test to identify feet at high risk of ulceration rather than just neuropathy [12, 22].

The prevalence of foot insensitivity in our study was higher in White Europeans compared to South Asians which is consistent with previous studies from other groups in the UK [23–25]. However, our results (Table 3) show that while foot insensitivity was associated with greater eGFR decline and greater progression to albuminuria in both South Asians and White Europeans, the between group differences were greater and statistically significant only in the South Asian group. The exact reason for this observation is not clear from this study, but one possible cause is that South Asians who develop foot insensitivity have more severe vascular disease than those with normal foot sensitivity in spite of foot insensitivity being less common in South Asians vs. White Europeans. Whereas, in White Europeans, there might be other factors at play contributing to the decline in eGFR such as obesity and OSA [10, 11].

In contrast to other diabetes related complications, in spite of improvements in glycaemia and BP control as well as RAAS inhibition, CKD leading to ESRD showed no signs of reducing in prevalence in a recent study from the US over a 20 year period [26]. Hence, it is important to identify patients who are at increased risk of eGFR decline in order to implement preventative strategies including intensive metabolic control. Currently, ACR is the main biomarker used in clinical practice to predict CKD and ESRD but 30% of patients still develop ESRD without the development of albuminuria [5, 6]. Free light chains have also been proposed as a marker of CKD and eGFR decline in patients with Type 2 diabetes, but they are expensive to perform and need further validation [27]. Our results demonstrate that simple, not expensive and routinely performed foot examination can identify patients at increased risk of eGFR decline.

Our study has also several strengths. It is the first to prospectively examine the relationship between foot insensitivity and eGFR as well as albuminuria and eGFR in patients with type 2 diabetes. We used simple, inexpensive, reproducible and routinely performed tests to predict eGFR changes in this study. The study-population was also well characterised with measurement of a wide range of demographic, clinical and biochemical variables allowing adjustment for a range of potential confounders. Furthermore, the study included patients of both genders and of both South Asian and White European ethnicity. In addition, we focused on patients relatively early in the course of CKD (as judged by baseline eGFR). This population would be a prime target for treatments that might slow the progression toward ESRD.

Our study has several limitations. We have used a single ACR measurement instead of 2 out of 3 measurements. However, the use of single, rather than multiple, ACR measurement has been used in previous studies and was shown to be adequate in epidemiological studies [17–19]. The relatively small sample size and the missing follow-up data for albuminuria, is a weakness. However, no differences in characteristics of participants versus patients lost to follow-up were observed. This was a relatively small, single centre study so that the associations described should be viewed as hypothesis generating. Further, larger multi-centre studies are now required to confirm whether the associations described are correct and in turn to suggest further studies that need to be done to look into mechanisms of the described associations. In addition, despite adjustment for multiple variables in the linear regression, we cannot rule out the presence of other confounders that need to be explored in future studies.

## Conclusion

In summary, patients with Type 2 diabetes and foot insensitivity are at increased risk of greater eGFR decline. Identifying these patients might offer an opportunity to intensify metabolic control and prevent the development of CKD. Future studies of larger sample size and longer follow up from multiple centres are needed to assess the diagnostic performance of our findings in predicting CKD development, and to compare the performance of the monofilament test with albuminuria.

## Abbreviations

ACR: Albumin creatinine ratio
BP: Blood pressure
CAN: Cardiac autonomic neuropathy
CKD: Chronic kidney disease
eGFR: Estimated glomerular filtration rate
ESRD: End stage renal disease
HbA1c: Glycated haemoglobin
IHD: Ischaemic heart disease
OAD: Oral anti diabetes treatment
OSA: Obstructive sleep apnoea
PVD: Peripheral vascular disease
RAAS: Renin–angiotensin–aldosterone
RRT: Renal replacement therapy
